# Peg Biology: Deciphering the Molecular Regulations Involved During Peanut Peg Development

**DOI:** 10.3389/fpls.2019.01289

**Published:** 2019-10-18

**Authors:** Rakesh Kumar, Manish K. Pandey, Suruchi Roychoudhry, Harsh Nayyar, Stefan Kepinski, Rajeev K. Varshney

**Affiliations:** ^1^Center of Excellence in Genomics and Systems Biology, International Crops Research, Institute for the Semi-Arid Tropics (ICRISAT), Hyderabad, India; ^2^Centre for Plant Sciences, University of Leeds, Leeds, United Kingdom; ^3^Department of Botany, Panjab University, Chandigarh, India

**Keywords:** abscisic acid, embryo abortion, peanut, gravitropism, phototropism, hormone cross-talk, miRNA-regulomics, molecular omics

## Abstract

Peanut or groundnut is one of the most important legume crops with high protein and oil content. The high nutritional qualities of peanut and its multiple usage have made it an indispensable component of our daily life, in both confectionary and therapeutic food industries. Given the socio-economic significance of peanut, understanding its developmental biology is important in providing a molecular framework to support breeding activities. In peanut, the formation and directional growth of a specialized reproductive organ called a peg, or gynophore, is especially relevant in genetic improvement. Several studies have indicated that peanut yield can be improved by improving reproductive traits including peg development. Therefore, we aim to identify unifying principles for the genetic control, underpinning molecular and physiological basis of peg development for devising appropriate strategy for peg improvement. This review discusses the current understanding of the molecular aspects of peanut peg development citing several studies explaining the key mechanisms. Deciphering and integrating recent transcriptomic, proteomic, and miRNA-regulomic studies provide a new perspective for understanding the regulatory events of peg development that participate in pod formation and thus control yield.

## Introduction

Peanut or groundnut (*Arachis hypogaea* L.) is one of the most important leguminous crops which is consumed all over the world in various forms. The nutritious seeds of peanut consist of up to 50% edible oil and about 30% protein, as well as several vitamins and minerals, and are used in major food products such as confectionery, peanut butter, peanut candy etc. It is noteworthy that the worldwide production of peanut has reached 43,982,066 tonnes, with the majority of this coming from Asia and Africa (FAO, 2016). To meet the growing demand, breeders have used cultivated gene pool as well as diploid ancestral species to develop varieties with high yield, resistant to devastating diseases and tolerant to abiotic stresses (Varshney et al., 2009; Varshney et al., 2013a).

Hundreds of thousands of angiosperm plant species have distinctive reproductive mechanisms that allow them to form aerial fruits harbouring seed. However, there are plants that have evolved to produce fruits beneath the soil. These species often exhibit a unique way of producing subterranean fruits, known as geocarpy, involving self-fertilizing subterranean-cleistogamous flowers developed on underground shoots, as observed in Vigna subterranean (Tan et al., 2010). The exception is peanut, a member of *Fabaceae*, which develops aerial cleistogamous flowers but subterranean geocarpic fruit (pods). These modifications have evolved to provide adaptations for a particular environment and are key strategy for reproduction for each plant type. The key adaptive trait in peanut is the formation of a structure known as the peg. The peg develops after double fertilization due to elongation of intercalary meristematic cells present at the basal region of ovary. The peg has the capacity for positive gravitropism to move towards and penetrate the soil to form subterranean pods. Interestingly, failure of peg penetration into the soil leads to abortion of developing embryo and thus incur yield loss. Therefore, production of peanut is critically dependent on generation of pegs and their penetration into the soil (Luz et al., 2011). These unique features of peanut have attracted physiologists and researchers to explore the genetic control driving the positive gravitropic growth of the peg (Moctezuma, 2003; Chen et al., 2016a).

Over the last 3,500 years, peanut has been domesticated for food by selecting certain characteristics, which led to greater yield, especially increased numbers of seeds and fruits (see Varshney et al., 2017). Further selective breeding has generated varieties with enhanced yield through improved translation of light to photosynthetic product. One of the most important advances in peanut domestication was the selection of variants, which can partition appropriate amount of photosynthate to pegs along with their viability and number (Luz et al., 2011). Therefore, identifying unifying principles for the genetic control of peanut peg development is fundamentally important to improve crop yield. New evidence has largely originated from the recent molecular-omics approaches deployed to understand the molecular mechanisms involved during peg development (Chen et al., 2016a; Chen et al., 2016b). This research provided evidence about interactions of genes, proteins and regulatory noncoding RNAs involved in peg — gravitropic responses, phototropism, embryo abortion, calcium signaling, hormone cross-talk, and photomorphogenesis, which can improve performance and productivity. Recent availability of the genome assemblies of the cultivated peanut (Bertioli et al., 2019; Chen et al., 2019; Zhuang et al., 2019) is expected to advance knowledge further in this important area.

This review provides an update on the progress being made in the molecular aspects of peg development, and draws the attention of plant biology researchers to explore peanut peg as model for gravitropism, photomorphogenetic, and developmental studies.

## Peg: A Specialized Organ in Peanut

Peanut has a unique mechanism to embed fertilized ovary of flower into the ground through specialized organ known as the peg or gynophore. Peg is a tube like structure which is formed after successful fertilization in flowers. After fertilization, the growth of the embryo remains arrested, and the intercalary meristematic cells beneath the ovary (part of the short stalk or pedicel) start to divide rapidly which leads to the formation of peg (Moctezuma, 1998). Therefore, some botanists and researchers also prefer to use term “gynophore”. The peg elongation stops when it buries the embryo into the soil, and afterwards the growth of embryo resumes.

Peanut subterranean pegs are crucial for the growth of the developing pod as well as the plant. Webb and Hanse (1989) demonstrated that the subterranean pegs mediated plant survival through excision of main stem from the root system. The subterranean peg develops root hair-like structures that facilitate absorption of adequate amounts of moisture and nutrients from the soil required for the overall plant growth. Further, the peg absorbs nutrient and moisture from soil and develop fruits in a soil but not in the water (Harris, 1949; Van der Volk, 1914). Thus, the peg is a unique evolutionary adaptive structure that facilitates peanut reproduction and dispersion within close proximity of the parent plant through less inter-specific competition. This ecological adaptation significantly increases fitness by extending the duration of pod development and protecting pods from unfavourable abiotic conditions above ground. However, this restricts the genomic fluidity of peanut germplasm causing dilution of genetic variation; arisen mainly because of self-fertilization, prolonged cultivation, and local adaptation.

The soil profile is important for pegging and pod development. Calcium (Ca) and sulphur is beneficial in the pegging zone as its deficiency in the soil can inhibit the swelling of peg and pod formation (Harris, 1949). The study on establishing role of Ca and nitrogen (N) influencing flowering, peg or pod production showed synergistically influence of Ca and N on peg development but not pod development (Taylor and Moshrefi, 1987). Another study demonstrated the role of soil during pegging and pod development, and reported loam soil more suitable for peg swelling and pod development, as well as yield formation over clay and sandy type of soil (Zhao C. et al., 2015). The loam soil provides moderate aeration and water along with fertility retention, a favourable condition for plant and peg growth, and pod development (Zhao C. et al., 2015). The optimal growth of subterranean peg and pod require slightly acid soil pH of 6.0 to 6.5, but a range of 5.5 to 7.0 is acceptable.

## Behaviour, Morphology, And Anatomy Of Peg

**Figure 1:**
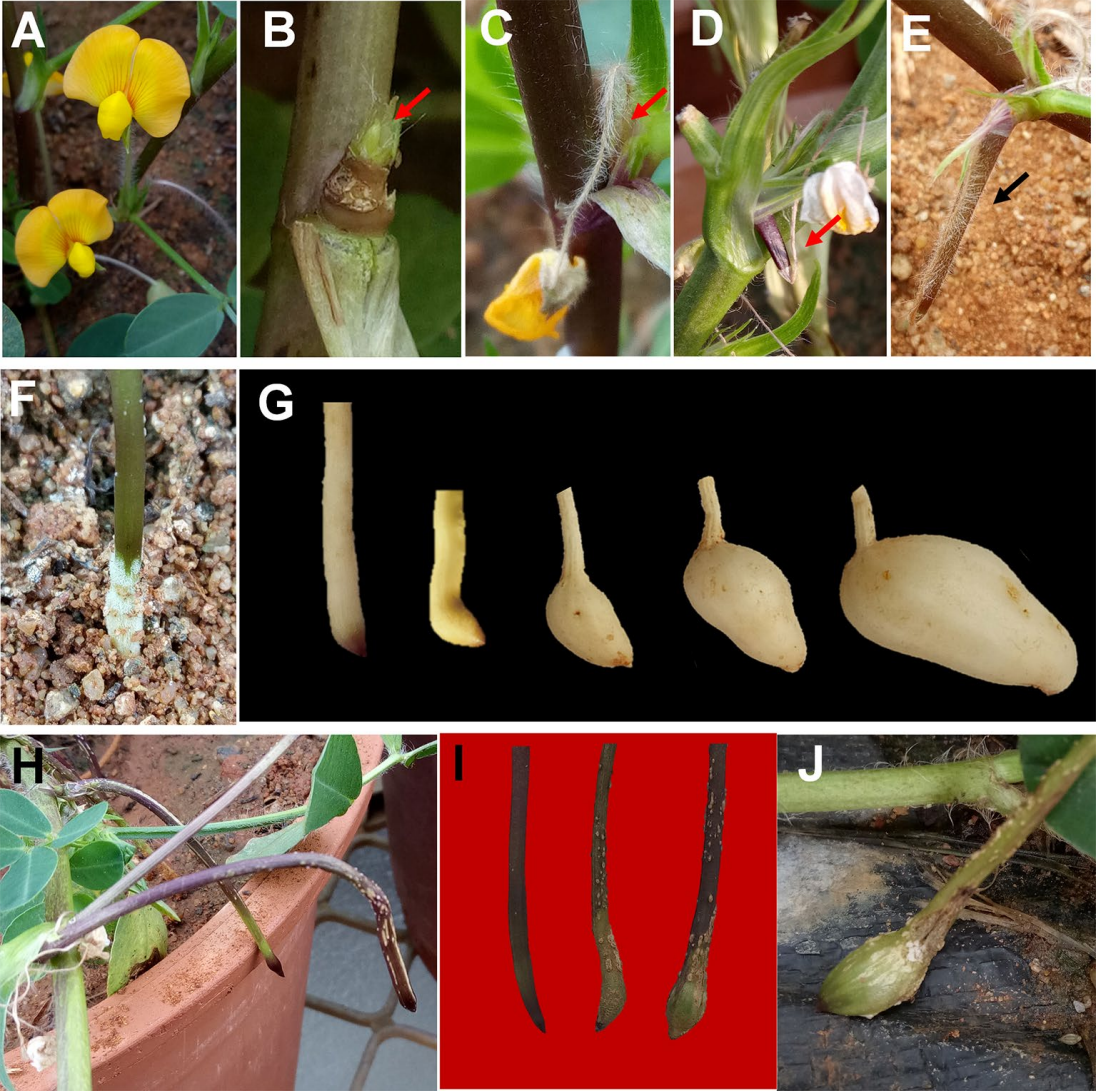
Peg development and formation of aerial pod in peanut. **(A)** flower; **(B)** emasculated flower ovary at the base of gynophore; **(C)** emergence of peg from dehisced flower; **(D**–**E)**, peg bending towards gravity; **(F)** peg penetration into the soil with whitish hairs at the base of soil penetration; **(G)** morphological changes into a peg after soil penetration; **(H)** abortion of aerial peg due to failure of soil penetration; **(I**–**J)** development of aerial pod under water deficit condition. Arrow is used to highlight the specified content of respective images.

**Figure 2:**
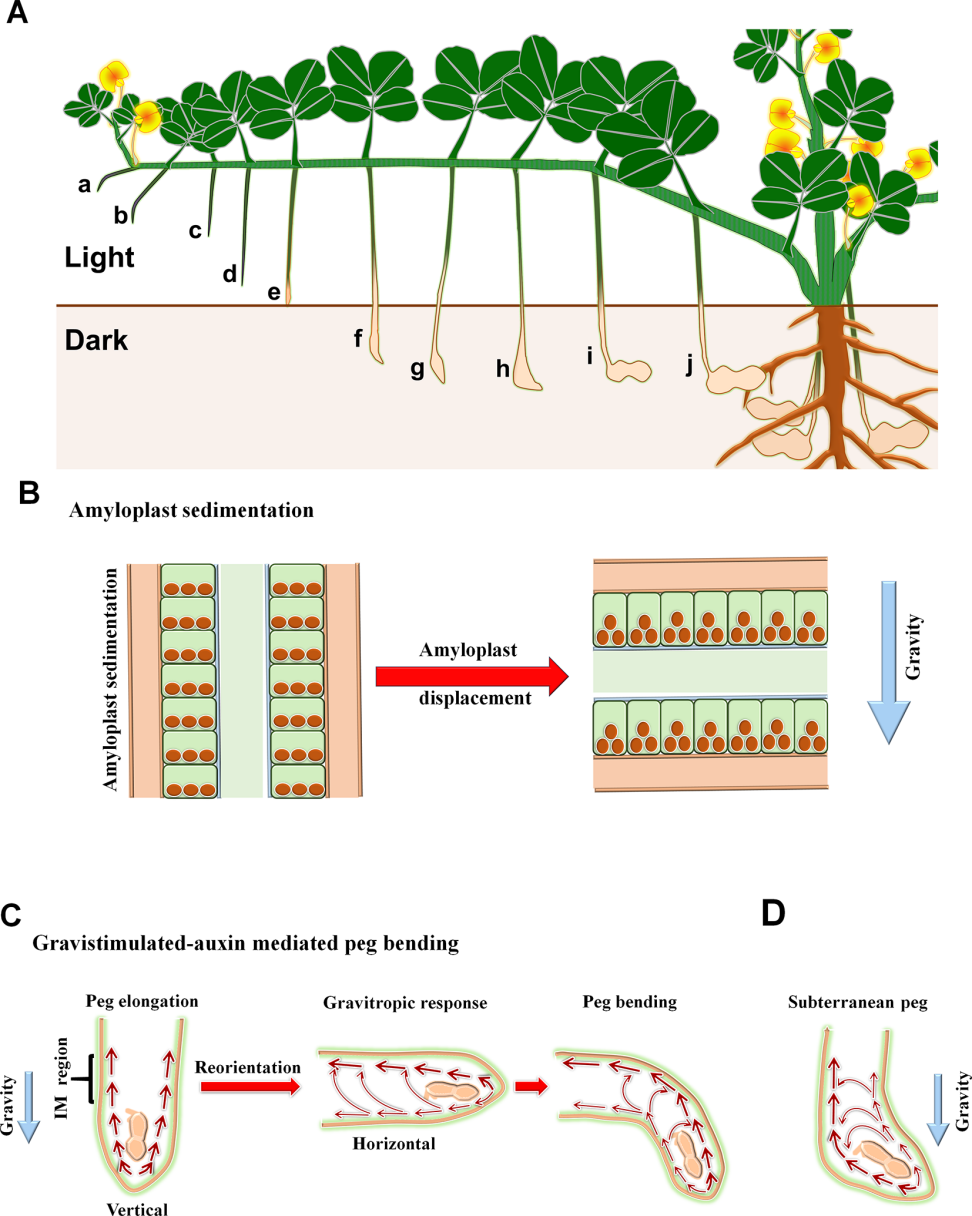
Representation of morphological and physiological changes associated with peg development and pod initiation. **(A)** Pictorial representation of pegging. a–b, gravitropic response; c–d, peg elongation; e, peg touching soil which impact mechanical stimulus; f–h, peg tip reorientation against gravity; i–j, pod swelling and seed setting; **(B)** Sedimentation and displacement of amyloplast during peg elongation (vertical position, left panel) and gravity induced bending (horizontal position, right panel); **(C)** Distribution of auxin in peg during elongation and gravitropic bending, and **(D)** agravitropic bending of subterranean peg. Thickness of arrow indicates the abundance of auxin.

Positive gravitropism is a peculiar feature of the peg. In peanut, sucessful fertilization results in initiation of the peg which carries mitotically arrested cells of embryo (Moctezuma, 2003). The emerged peg senses gravity and bends downward. Initially the emerged aerial peg does not show any changes in the developing embryo as mitotic division is arrested with the embryo remaining at the proembryo stage. Later, the intercalary meristem residing at the base of the ovary divides rapidily resulting in peg elongation and implantation of the arrested embryo into the soil. Notably, after the peg tip penetrates the soil vertically, it reorients horizontally and perceives signals that prompts the resumption of embryo cell division, facilitating geocarpic pod development (**Figures 1** and **2A**). The perception of mechanical stimulus and darkness is essential for transformation of the peg into a pod. Without these signals the embryo aborts (Moctezuma, 2003), leading to formation of hard lignified green-aerial pods, which can also be observed under water deficit conditions (**Figure 1**).

The anatomy of the peg typically resembles the shoot (Moctezuma, 1998). However, the anatomy of the unfertilized peg, compared to the fertilized peg, varies greatly, with the former lacking starch granules (Moctezuma and Feldman, 1998, Moctezuma and Feldman, 1999b; see below). The aerial peg consists of multicellular trichomes of five to six cells in length, of which the terminal cell elongates compared to the first four to five proximal cells (Webb and Hanse, 1989). After the peg penetrates the soil, unicellular hairs similar to root hairs develop abundantly on the subterranean peg surface. Interestingly, while the anatomy of peg is related to stem, its behaviour and functionality changes after soil penetration, and resembles a root, especially just after soil penetration (Moctezuma, 2003). The exclusive features of aerial peg are smooth epidermis and the presence of numerous stomata and lenticels, gradually disappear and become obscured by tufts of hairs present in subterranean peg (Webb and Hanse, 1989).

The aerial peg is self-sufficient for energy production because it possesses active photosynthetic physiological structure and machinery (Zhao C. et al., 2015). Additionally, the considerable photosynthetic activity of the sub-epidermal parenchyma tissue, the presence of stomata, and high starch content are consistent with the photosynthetic properties of aerial peg (Webb and Hanse, 1989; Zhao C. et al., 2015). For instance, proteome mapping at peanut reproduction and pegging stages identified expression of approximately 34 photosynthesis-related proteins in the aerial peg such as photosystem II type I chlorophyll a/b-binding proteins, oxygen evolving enhancer protein 1/2, rubisco activase, plastocyanin, etc., representing a sub-set of core proteins involved in photosynthesis (Zhu et al., 2013; Zhu et al., 2014). The number of these photosynthetic proteins was drastically reduced in subterranean peg/pod. In contrast to these photosynthetic proteins, other multiple energy metabolism related proteins such as glycolytic pathway proteins — fructose bisphosphate aldolase and triosephosphate isomerase were also identified in the subterranean peg. Similarly, proteome of developing peg revealed large-scale expression profile changes in the proteins related to fatty acid pathway: biosynthesis, elongation, and formation of unsaturated fatty acids (Zhu et al., 2014). This study also highlighted the differential expression of proteins involved in the biosynthesis of auxin, ethylene, and gibberellin and hormone signaling during pegging. Additionally, another recent proteome study emphasizes involvement of brassinosteroid during peg development (Zhao C. et al., 2015). These findings strengthen and support the existing knowledge about hormone signaling involved in peanut peg biology.

## Peg Gravitropism: Deciphering the Molecular Pathways

**Figure 3:**
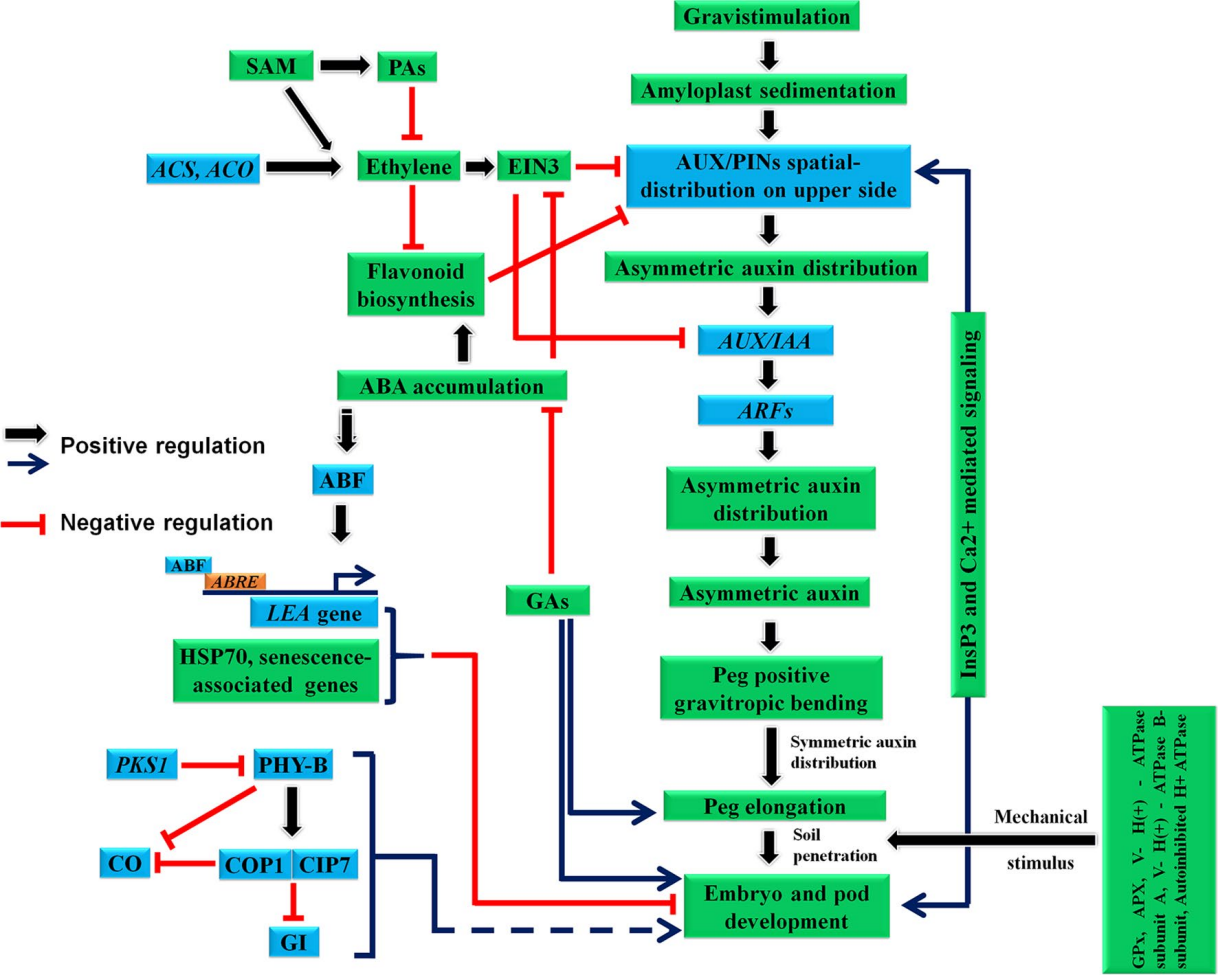
Probable molecular regulatory mechanism involved during peg development, gravitropic bending, elongation and pod development. ABA, abscisic acid; ABRE, ABA-responsive element; ABF, ABRE-binding factors; ACO, ACC oxidase; ACS, ACC synthase; APX, Cytosolic ascorbate peroxidase; COP1, E3 ubiquitin–protein ligase constitutive photomorphogenic 1; CIP7, COP1-interacting protein 7; CO, constans; EIN3, ethylene-insensitive3; GA, Gibberellic acid; GI, gigantea; GPx, glutathione peroxidase; IM,; LEA, late embryogenesis abundant; PAs, polyamines; PHY-B, phytochrome; PKS1, phytochrome kinase substrate 1; SAM, S-adenosylmethionine; V- H(+) - ATPase subunit A, Vacuolar - H(+) - ATPase subunit A; V- H(+) - ATPase B-subunit, Vacuolar - H(+) - ATPase B-subunit.

After fertilization, the week-old peg becomes gravitropic, a response involving multiple mechanisms. In this section, we have discussed different processes involved during peg gravitropic response (**Figures 2** and **3**).

### Amyloplast Accumulation and Spatial Distribution

Soon after fertilization, the previously agravitropic peg starts to accumulate amyloplasts and respond to gravity (Chen et al., 2016a). As in other gravicompetent tissues in the root and shoot, these starch-rich amyloplasts, that are denser than the cytoplasm, act as statoliths by sedimenting according to gravity (Roychoudhry and Kepinski, 2015). This gives the peg information on its orientation in the gravity field and hence the direction in which tropic growth must occur to reach the soil. Notably, the unfertilized peg has neither visible amyloplasts nor responds to gravity (Moctezuma and Feldman, 1998). Therefore, there is a strong correlation between amyloplast development and competence of the peg to bend with the gravity vector. The amyloplasts are located in the starch sheath in the apical region (2–8 mm) of the peg tip which includes the main elongation zone near intercalary meristem (Moctezuma and Feldman, 1998). These cells are therefore the gravity-sensing cells or statocytes of the peg. Usually, amyloplasts are sedimented at the apical surface of the peg statocytes (relative to the peg tip) in vertically-oriented peg; however following the reorientation of the peg to the horizontal the amyloplasts come to rest on the lateral wall of the statocyte i.e to the new lower surface of the starch sheath cells just before the formation of gravitropic curvature (**Figure 2B**; Moctezuma and Feldman, 1998; Moctezuma and Feldman, 1999b). In the root, amyloplast sedimentation is required for full gravitropic response as demonstrated in studies with *Arabidopsis* starchless mutants such as *pgm1-1* and *TC7* whose empty amyloplasts do not readily sediment (Kiss et al., 1989; Wolverton et al., 2011). Further, in both maize and wheat, where the root is decapitated, gravitropic response only resumes upon the regeneration of statocytes and the development of new amyloplasts (Barlow, 1974). In peanut, the application of exogenous gibberellic acid (GA) and kinetin was able to destarch the peg resulting in starchless amyloplasts and an almost complete loss of gravitropic response (Moctezuma and Feldman, 1999b). Together, these studies provide a very strong evidence for the amyloplast assisted peg gravitropism.

### Asymmetric Spatio-Distribution of Auxin Precedes Gravistimulated Bending

In both shoot and root, gravity perception causes asymmetric redistribution of auxin, which results in bending away or movement towards gravity vector, respectively (Roychoudhry and Kepinski, 2015; Harmer and Brooks, 2018). In peanut, the peg produces the auxin indole-3-acetic acid (IAA) in the tip region, which distributes basipetally in a polar manner assisting gravitropism (**Figure 2C**; Moctezuma and Feldman, 1999a). Consequently, decapitation of the peg tip or in the presence of auxin transport inhibitors, the peg losses its geotropic capacity (Moctezuma and Feldman, 1999a). In the case of decapitation, graviresponse can be restored by the application of exogenous IAA. The immunolocalization experiments have confirmed localization of IAA in the intercalary meristem, epidermis and cortex of elongation zone, and the area adjacent to the seed in vertically-growing peg (Moctezuma and Feldman, 1999a). Further, the placement of an aerial peg to the horizontal direction induces the accumulation of IAA in the upper epidermis and cortex region, with the consequent auxin concentration gradient between the upper and lower halves of the peg driving downward gravitropic growth (Moctezuma and Feldman, 1999a; Moctezuma and Feldman, 1999b). Crucially, this gravity-dependent upward redistribution of auxin is the opposite of that usually associated with gravity response in the shoot (Roychoudhry and Kepinski, 2015). While it is clear that the requirement for this difference arises from the need to drive downward, rather than upward tropic growth, it is nevertheless an intriguing phenomenon because in current models of auxin transport-dependent gravitropic response the polarity of asymmetric auxin transport is the same as for amyloplast sedimentation, i.e. downwards. These observations also suggest that the response of the peg tissues to auxin in terms of cell elongation is the same as observed in other shoot tissues, with auxin promoting cell expansion rather than inhibiting it as observed in the root (Roychoudhry and Kepinski, 2015). A more profound insight into the spatio-temporal distribution dynamics of IAA was established by implanting an impermeable membrane barrier in the vertically-positioned peg, separating left and right halves of the organ that was later reoriented to the horizontal such that these halves became upper and lower (Moctezuma and Feldman, 1999a). In both cases, the radiolabelled-IAA signal was higher in the upper cortex compared to lower cortex, indicating that there is basipetal IAA transport away from the peg tip to the upper-side cortex, possibly arising from IAA synthesis in the inert embryo. This underlines the possibility for existence of two-auxin transport mechanisms during peg development and geotropic response: (a) polar basipetal auxin transport from tip to elongation zone facilitating peg elongation, (b) lateral transport of auxin from lower cortex to the upper cortex aiding gravitropic bending (**Figure 2C**).

Gravistimulation response increases cytoplasmic Ca2+ and changes pH of apoplast that induce cell wall acidification and increases extensibility, which changes polarity of auxin transport and the localization of PIN-FORMED (PIN) auxin efflux carriers (Zhang et al., 2011; Shih et al., 2015). During development of peanut peg and pod, auxin efflux carriers are also upregulated along with auxin-conjugate hydrolases, and many other auxin-induced proteins. These proteins have great influence on auxin-related physiological response (Zhao et al., 2015). In the *Arabidopsis* root, auxin efflux carriers PIN3, PIN7, and ALTERED RESPONSE TO GRAVITY1/2 (ARG1/2) proteins are involved in the gravity response in the columella statocytes. The polarity of PIN3 and PIN7 within the columella is gravity-dependent and upon gravistimulation, both proteins can rapidly become asymmetrically localised to the lower face of the statocyte (Kleine-Vehn et al., 2010; Rakusová et al., 2011). ARG1 is involved in the PIN3 polarization which results in a subsequent asymmetric redistribution of auxin (Harrison and Masson, 2008). These findings highlight the parallels between gravitropic response in both the peanut peg and all other gravitropic organs and indicate that asymmetric distribution of auxin underlies the gravitropic response of peanut pegs.

### Auxin-Ethylene Co-Action During Gravitropic Response

Interaction of ethylene with auxin during development is well surveyed *in planta* (Pozhvanov et al., 2017). In general, ethylene acts antagonistically to auxin (and auxin signaling pathways); therefore it has a negative impact on both root and shoot gravitropism (Hu et al., 2017). In peanut, development of peg involves an influx of auxin corroborated with slow evolution of ethylene (Shlamovitz et al., 1995). Notably, ethylene levels tend to become elevated during peg burying or post-burying, while auxin levels become depleted (Shlamovitz et al., 1995). This phase includes friction with soil, peg reorientation towards the horizontal (agravitropic movement) and the swelling of peg and pod enlargement. After this phase, ethylene production declines (Shlamovitz et al., 1995). Further, when the peg bends sideways during early fruit formation (pod), IAA accumulation increases at the lower portion to facilitate its growth as well as upward bending (Moctezuma and Feldman, 1999a; Peng et al., 2013). Ethylene is known to enhance basipetal auxin transport by inducing AUX1 and PIN2 transcripts which reduces root gravitropism (Růžička et al., 2007; Negi et al., 2008). Additionally, treating roots with ACC (a precursor for ethylene in its biosynthesis pathway) supresses root gravitropism through an ETR1 and EIN2-dependent pathway by regulating basipetal auxin transport (Buer et al., 2006). Further, strong evidence for ethylene–auxin interactions during gravitropism comes from Arabidopsis dominant mutations of AXR2/IAA7 and AXR3/IAA17 genes which displayed abrupt gravitropic curvature and reduced ethylene sensitivity during gravitropic response (Timpte et al., 1995). Taken together, these findings suggest a strong correlation between auxin and ethylene during peg gravitropism (**Figure 3**). Therefore, we propose that auxin–ethylene co-action is required during peg gravitropic responses in peanut. Additional focussed and in depth studies are required to gain further mechanistic insights in this perspective.

### Non-Hormone Encoding Genes Involved in Gravitropic Response

Recently, an *in-vitro* culture system was used to examine peg gravitropic response (Li et al., 2013). This study underlined the role of several non-hormone encoding genes that could play important roles in peanut gravitropic response. Interestingly, gravity stimulus induced altered expression of several of these proteins; among which 13 proteins were prominent and consistent with having a role in mediating the gravitropic behaviour. These include three members of a germin-like protein subfamily, two class III acidic endochitinases, two Kunitz trypsin protease inhibitors, a pathogenesis related class 10 protein, a voltage-dependent anion channel, a chloride channel, an Ara h 8 allergen isoform 3, γ- carbonic anhydrase-1, and glycine-rich RNA-binding proteins, along with a hormone-related protein (gibberellin receptor GID1). Interestingly, among these 13 proteins, six of them have roles characterised in plant defence pathways, suggesting that these proteins may play dual roles in defence and gravitropic responses. Previously, a similar finding was reported in *Arabidopsis*, where hyper-gravity and mechanical stimulus caused differential expression of defence-related genes (Martzivanou and Hampp, 2003; Kimbrough et al., 2004). It is noteworthy that pathogenesis-related class 10 protein was found to be expressed in the endodermis region of root (Kim et al., 2008), which is also known as gravity sensing region (Haswell, 2003). Recently, a proteomic study of pine and poplar has also suggested the role of class III acidic endochitinases during gravitropic response. These genes are associated with carbohydrate metabolism, and are also storage proteins (Azri et al., 2009; Herrera et al., 2010), residing at the starch granules of the amyloplast, which is known to be involved in sensing the direction of gravity to promote downward gravitropic curvature (Lv et al., 2011). In plants, carbonic anhydrases are also involved in carbohydrate metabolism and photomorphogenesis (Wang et al., 2012). Voltage-gated anion channel and chloride channel proteins play important role in nutrition uptake, metabolism and signal transduction (Kollist et al., 2011). For example, their role in auxin transport has been also described in *Arabidopsis* where they regulate auxin-mediated hypocotyl growth (Thomine et al., 1997; Iino et al., 2001), suggesting that they may play an important role during peg gravitropism.

### Metabolites Association With Gravitropism

Metabolites regulate key functions during plant growth, development and reproduction (Kumar et al, 2017; Kumar et al., 2018), and they are involved in both types of processes: biochemical and physiological. In peanut, metabolites play a significant role during pod development. For instance, the evolution of low level of ethylene in the aerial peg was also correlated with the occurrence of high levels of polyamines (PAs) and flavonoids in the developing aerial peg, which decline in subterrenian peg and pod after soil penetration (Savithramma and Swamy, 1989; Moctezuma, 2003). Notably, PAs are negative regulators of ethylene biosynthesis and are known to inhibit auxin-induced ethylene in leaf and fruit (Apelbaum et al., 1981; Kushad et al., 1988). Both ethylene and PAs share a common precursor: S-adenosylmethionine (SAM), thus when higher level of PAs are synthesized, ethylene level declines. Interestingly, one of gravi-responding genes is predicted to encode an isoform of enzyme acting on SAM: carboxyl methyltransferase which transfers methyl groups from SAM to other molecules (Kimbrough et al., 2004). Similarly, biosynthesis and accumulation of flavonoids in the aerial peg co-incides with low/basal levels of ethylene evolution, and high levels of auxin and ABA. A previous study has shown that gravitropic response induces flavonoid accumulation in the Arabidopsis root tip (Buer and Muday, 2004). The abundance of protein chalcone isomerase D (a key enzyme for flavonoid biosynthesis) in the aerial peg also provides an insight into the role of flavonoids during peg gravitropic movement (Sun et al., 2013). Interestingly, flavonoids were identified as the negative regulators of auxin transport through inhibition of the auxin efflux carriers, which alter the auxin gradient, facilitating asymetric auxin distribution and accumulation gravitropic response (Jacobs and Rubery, 1988). Recently, a correlation between flavonoid biosynthesis and ethylene–auxin levels during root gravitropic reponse and elongation was demonstrated in Arabidopsis (Buer et al., 2006). Therefore, flavonoids are key molecules which can impact the peg development and tropism *via* hormones - ethylene and auxin (**Figure 3**). Plausibly, a similar mechanism may co-exist in the peg which includes assimilation of high level of favonoids in the aerial peg whose levels subsequently decline after peg penetrates the soil, concomitant with ethylene burst. These mechanisms are recenlty supported by investigation of aerial and subterennean peg for examination of genes and proteins expression which are associated with the biosynthesis of ethylene and flavonoids, auxin signaling and gravitropic response.

### Lipid Signaling Might Play Significant Role During Peg Development and Gravitropism

Lipids are associated with gravitropism and involved in the gravity perception through calcium signaling and auxin redistribution (Smith et al., 2013). The lipid derived inositol triphosphate (InsP_3_) plays a crucial role in the gravitropic response through Ca2+ (Smith et al., 2013). Arabidopsis transgenic plants constitutively expressing InsP-5-ptase enzyme had reduced levels of InsP_3_, which results in reduced bending of hypocotyls, roots and stems upon reorientation (Perera et al., 2008). InsP_3_ has a potential to trigger accumulation of intracellular Ca^2+^, which plays a significant role in gravitropic response (Yang and Poovaiah, 2003).

The lipid-mediated auxin redistribution is a highly regulated complex process involving both auxin efflux (PIN/ABCD) and influx carrier (AUX/LAX) transporter proteins (Smith et al., 2013), which were found expressed in the developing peanut peg through transcriptomic and proteomic approaches. More recently, InsP_3_ and Ca^2+^ mediated signaling has been demonstrated during PIN protein localization, and a defect in the inositol-5-P results in altered PIN2 localization (Zhang et al., 2011). A dual role of Ca2+ signaling has been elucidated during development of peanut peg and additionally also during gravitropism-related auxin distribution and amyloplast sedimentation (Moctezuman and Feldman, 1996; Moctezuma and Feldman, 1999a;Moctezuma and Feldman, 1999b; Yang et al., 2017). The development of peg and pod involves several genes involved in lipid metabolism and signaling such as phospholipid transporters, phospholipid-transporting ATPase, lipid kinase, sterol binding protein, calcium-dependent lipid-binding-like protein, phosphatidylinositol 3- and 4-kinase, myo-inositol transmembrane transporter, phosphatidylinositol phosphatase, inositol bisphosphate phosphatase, etc. (Xia et al., 2013). For ground-based studies, information of these components in detail and correlation of physiological studies by observing mutants could be useful to gain detailed knowedge of lipid mediated gravitropic response in peanut peg.

## Role of Heat Shock Proteins During Peg Development

Heat shock proteins (HSPs) are broadly known to be involved in stress responses; however, their roles in gravitropic responses are less explored. In *Arabidopsis*, evidence for HSPs mediated gravitropism comes from a *rha1* mutant that shows resistance to auxin and exhibits altered root bending in response to a gravitopic stimulus (Fortunati et al., 2008). A recent study has uncovered a new function of HSPs as transcription factors, therefore, they play a significant role in various processes including phosphorylation of proteins as well as interaction with calmodulin — a calcium signaling protein (Park and Seo, 2015). In peanut, transcriptome and proteomic analysis of aerial and subterranean peg revealed the expression of more than 100 HSPs-related unigenes associated with gravitropic response, mechanical stimulus and light and dark regulation. Among these, 13 HSPs were proposed to be key controllers of gravitropic response (Xia et al., 2013; Zhao C. et al., 2015). These results provide new insights into peanut biology as HSPs are also known to be associated with embryo abortion (Fu et al., 2002; Hsu et al., 2010). Earlier, the role of HSPs in embryo development was demonstrated in maize and *Arabidopsis* (Fu et al., 2002; Hsu et al., 2010). In peanut, HSP-70 is highly expressed in the aerial peg, while its expression is attenuated in the subterranean peg during swelling and pod formation (Xia et al., 2013). Thus, there is a scope to study and confirm if the association of HSPs with embryo abortion in peg is similar to previous findings reported in the maize and *Arabidopsis*. Recently, protein modifying- and degrading enzyme threonine endopeptidase was reported in the developing peg and pod, suggesting its critical role during embryo development and potentially premature embryo abortion (Zhu et al., 2013). In barley, threonine endopeptidase causes degeneration of aleurone cells (Swanson et al., 1998); whereas, it is associated with floral organ senescence in daylily (*Hemerocallis* spp) (Guerrero et al., 1998), thus plays a central role in the developmentally associated programmed cell death (Beers et al., 2000). Hence, further detailed studies in this regard would be useful to achieve high yield *via* improved pegging and low embryo degeneration.

## Diverse Function of Hormones During Peg And Pod Development

### GA and Cytokinin Have Major Effects on Cell Elongation and Division

The developing peanut peg also produces a significant amount of GA to promote the cell elongation that ultimately facilitates peg elongation. Afterwards, GAs concentration declines once the peg penetrates the soil and buries (Shushu and Cutter, 1990). It was demonstrated that a combination of GA_3_ and auxin could restore peg growth in excised pegs as compared to the intact ones (Shushu and Cutter, 1990). However, unlike auxin, which affects only young cells of peg, GA_3_ can promote cell elongation across the entire length of peg (Shushu and Cutter, 1990). In contrast, cytokinin regulates cell division of the juvenile peg structure. As described earlier, during peg development cytokinin accumulates at the early stages to facilitate cell division (Moctezuma, 2003). Interestingly, kinetin (a type of cytokinin)-induced peg elongation in the dark, does not occur in decapitated pegs, suggesting that auxin and cytokinin interact during peg elongation (Shushu and Cutter, 1990). In contrast, application of GA_3_ restores elongation of kinetin treated decapitated peg, suggesting that GA_3_ may act by elongating the newly dividing cell, facilitated by kinetin application.

### Ethylene and Triple Response Phenotype of Peg

As described earlier, ethylene levels tend to become elevated during peg burying or post-burying, and the white peg developed after soil penetration demonstrates the “triple response” phenotype shown by etiolated pea seedlings exposed to higher ethylene: thick and short hypocotyl, radial swelling, and horizontal growth habit (Shaharoona et al., 2007). Ethylene also regulates cell division by regulating a gene encoding microtubule-stabilizing protein WAVE-DAMPENED2-LIKE5 (WDL5), a member of the WAVE-DAMPENED2 (WVD2) protein family, which reorganizes cortical microtubules during cell elongation (Sun et al., 2015). Interestingly, overexpression of *WVD2* in *Arabidopsis* also results in “triple response” phenotype in seedlings (Yuen et al., 2003). In peanut, role of microtubule-associated protein has been reported through combined transcriptome and proteome approach (Zhao C. et al., 2015). Additionally, Shlamovitz et al. (1995) demonstrated that application of the ethylene inhibitors aminooxyacetic acid and silver thiosulphate significantly affect the pod growth without altering the percentage of total pod formation (Shlamovitz et al., 1995). It is, thus, plausible that peanut peg maintains lower ethylene level in both the aerial peg and subterranean pod to facilitate cell division and elongation suggesting that a basal level of ethylene might be required to maintain normal cell division and elongation of developing peg and the pod swelling and elongation.

### ABA Directs the Embryo Growth of Developing Peg: Embryo Development and Abortion

Abscisic acid (ABA) controls cell division and elongation of a developing embryo (Da Silva et al., 2008). Therefore, ABA-deficient mutants have increased seed size and weight due to increased cell numbers in embryo (Cheng et al., 2014). In peanut, aerial peg exclusively produces high level of ABA, which progressively decline after the peg penetrates the soil and during pod development (Shlamovitz et al., 1995). Ziv and Kahana (1988) have evaluated the response of excised embryo in dark and found that embryo development was arrested by the exogenous application of ABA. Therefore, it cannot be ruled out that high levels of ABA found in aerial peg arrest the cell division of the developing embryo. Therefore, it is possible that in light conditions, the aerial peg maintains high level of ABA to arrest embryo growth. However, in the dark, a signal is perceived for resuming embryo growth *via* supressing ABA levels.

Interestingly, the discovery of high levels of ABA also supports the high levels of anthocyanin content of the aerial peg, which decline in the subterranean conditions correlating with low ABA levels because ABA known to regulate the anthocyanin content (Ferrara et al., 2015). As described earlier, desiccation/loss of soil moisture can induce formation of aerial pod, which provides another correlation with ABA levels, as ABA production is induced during drought stress (Kumar et al., 2017; Kumar et al., 2018). Further, increase of ABA beyond certain level can induce swelling of root tip in sorghum (Kannan and Shaikh, 1986), suggesting that it is likely to play a similar role in the aerial pod developed in peanut. Together, combining two previous observations we conclude: a) high level of ABA can cause tip swelling, and b) ABA negatively regulates the embryo development, suggesting that a shift in the ABA level in the developing peg is required for the embryo maintenance and proper development of pod or else it can induce aerial pod formation.

Recently, a comprehensive analysis of aerial and subterranean pod transcriptome reported three candidate genes, which could be responsible for embryo abortion in the aerial peg (Zhu et al., 2014). Among them, two were putative senescence-associated genes while the third was the *late embryogenesis-abundant* (*LEA*) gene all of which were dramatically upregulated in the aerial young pod. Additionally, *LEA* genes are highly expressed during late embryogenesis in *Arabidopsis*, wheat and cotton (Battaglia and Covarrubias, 2013), and are required for normal seed development (Manfre et al., 2006; Manfre et al., 2008). Senescence-associated genes are known to be involved in leaf senescence and fruit yield *via* hormone homeostasis (Gepstein et al., 2003; Kim et al., 2011; Lira et al., 2017). LEA proteins are highly conserved (Gao and Lan, 2016) and provide tolerance against drought/desiccation, heat and salt stress (Shih et al., 2008; Gao and Lan, 2016). Promoters of LEA encoding genes contain abscisic acid response elements (ABREs) and/or low temperature response (LTRE) cis-acting regulatory elements (Hundertmark and Hincha, 2008). Studies that explored the legume genome sequence identified *LEA* genes in *Phaseolus vulgaris, Glycine max, Medicago truncatula: Lotus japonicas, Cajanus cajan*, and *Cicer arietinum* (Varshney et al., 2012; Varshney et al., 2013b), and their role in legumes are anticipated to be same (Battaglia and Covarrubias, 2013).

### Light–Dark Regulation During Peanut Peg Development and Pod Swelling

In plants, photoreceptors percieve light and translate them into signals controlling various functions including phototropic reponse and reproduction (Gupta et al., 2014). In fact, an extended exposure of light can reduce the flowering and peg numbers as well as pod formation by limiting reproductive development of peanut (Quamruzzaman et al., 2018). Photoreceptor phytochromes play a cenral role in photomorphogenesis and are also likely to be involved in gravitropism. It was found that far-red and darkness can induce pod development by supressing peg elongation, which suggests that phytochromes may control peg and pod development (Moctezuma, 2003). Further, darkness induces loss of flavonoids *via* reduced expression of gene *chalcone synthase* (*CHS*), a key enzyme in the flavonoid biosynthesis pathway (Xia et al., 2013), which facilitates lignin development in the developing pod *via* diverting substrate to lignin biosynthesis.

### The Role of Light Signaling Genes

Movement of aerial peg from light to dark (subterranean) induces several light responsive molecular changes. Aproximately 27 years back, Thompson et al. (1992) demonstrated the presence of phytochrome in the peanut peg. Their results described the role of light signaling during pod development, because phytochrome was exclusively detected only in the subterranean peg ovule, and its accumulation was gradually increased in the growing embryo. This result was supported by a recent discovery which showed decreased level of phytochrome kinase substrate 1 (PKS1) transcript in subterranean peg and pod (Chen et al., 2013). PKS1 is an important component of phytochrome mediated signaling and could be involved in phosphorylating phytochrome, and induced in light treated hypocotyl of Arabidopsis (Lariguet et al., 2006) and together with PHOTOTROPIN 1, it modulates auxin transport during hypocotyl phototropism (Tsuchida-Mayama et al., 2010). Another key light signal transduction gene, CONSTANS (CO) gets upregulated in subterranean peg (Chen et al., 2013). However, the CO protein is under tight control of ubiquitin-mediated protein degradation, and is degraded through ubiquitination in the dark (Valverde et al., 2004). Similarly, circadian clock and phytochrome signaling gene GIGANTEA (GI) is downregulated in dark-grown subterranean pegs and pods (Chen et al., 2013). Further, the expression of ubiquitin ligase COP1 and COP1-interacting protein 7 (CIP7) was drastically decreased in dark-grown gynophores. In Arabidopsis, CIP7 protein physically interacts with COP1, a negative regulator of photomorphogenesis — in the dark COP1 localizes from the cytoplasm to the nucleus for repression of light regulated genes (Yamamoto et al., 1998). Recently, Chen et al. (2016b) identified expression of aproximately 245 genes associated with light signaling and photomorphogenesis including several which have been well characterized in Arabidopsis. Many of them were enriched within subterranean peg highlighting their role in pod development. Further, darkness induces loss of flavonoids *via* reduced expression of gene *chalcone synthase* (*CHS*), a key enzyme in the flavonoid biosynthesis pathway (Xia et al., 2013), which facilitates lignin development in the developing pod *via* diverting substrate to lignin biosynthesis.

A recent proteomic study revealed that transition from light to dark conditions severely reduce the expression of eight proteins in the subterranean peg, mostly involved in light signaling, photosynthesis and stabilization of light energy, energy metabolism and maintenance of cell structure (Zhu et al., 2013). This group includes WD40 domain proteins, oxygen-evolving enhancer protein1, light-harvesting chlorophyll a/b-binding protein, ATP synthase subunit and glycine-rich RNA-binding protein (**Table 1**). In *Arabidopsis*, two WD40 domain containing proteins COP1 and SPA1 have been identified; COP1/SPA complex drives the phyA degradation, which is a key player in light signaling (Saijo et al., 2008).

**Table 1:** List of important genes regulating peg development and consequently affecting yield.

Sl. No.	Trait	Gene/protein identified during peg development	Reference
1	Photosynthesis and light signal transduction	Photosystem I reaction centre and chlorophyll a/b-binding protein	Xia et al. (2013), Zhu et al. (2013)
		Photosystem II type 1 chlorophyll a/b-binding protein	Xia et al. (2013)
		Photosystem II protein D1	Zhu et al. (2013)
		Photosystem II CP43 protein	Zhu et al. (2013)
		Oxygen-evolving enhancer protein 1 (OEE1)	Zhu et al. (2013)
		Oxygen-evolving enhancer protein 2 (OEE2)	Zhu et al. (2013)
		Plastocyanin	Zhu et al. (2013)
		Plastocyanin A	Zhu et al. (2013)
		Ribulose-5-bisphosphate carboxylase	Zhu et al. (2013)
		Rubisco activase	Zhu et al. (2013)
		Thylakoid lumenal 29.8 kDa protein	Zhu et al. (2013)
		Early light induced protein	Zhu et al. (2013)
		Phytochrome kinase substrate 1-like protein	Zhu et al. (2013)
		Phytochrome A	Zhao C. et al. (2015)
		Phytochrome B	Zhao C. et al. (2015)
		Phototropin	Xia et al. (2013)
		Constans	Xia et al. (2013)
		COP1	Xia et al. (2013)
		COP1 interacting protein 7 (CIP7)	Xia et al. (2013)
		Gigantea	Xia et al. (2013)
		Circadian clock-associated FKF1	Xia et al. (2013), Zhu et al. (2014)
		Putative early light induced protein	Zhu et al. (2014)
		DNA damage-binding protein 1b	Zhu et al. (2014)
		S-adenosyl-L-methionine dependent methyltransferase, predicted	Zhu et al. (2014)
		Root phototropism protein (RPT)	Zhu et al. (2014)
		Lipoxygenase	Zhu et al. (2014)
2	Gravitropism	ABC transporter ABCE.2	Zhao C. et al. (2015)
		ABC transporter family protein	Zhao C. et al. (2015)
		white-brown-complex ABC transporter family	Zhao C. et al. (2015)
		ABC transporter family protein	Zhao C. et al. (2015)
		multidrug/pheromone exporter MDR family ABC transporter family	Zhao C. et al. (2015)
		PDR-type ABC transporter 2	Zhao C. et al. (2015)
		Non-intrinsic ABC protein 6 (ATNAP6)	Zhao C. et al. (2015)
		DnaJ protein	Zhao C. et al. (2015)
		Microtubule-associated protein MAP65-1a	Zhao C. et al. (2015)
		heat shock protein 22	Zhao C. et al. (2015)
		heat shock protein 60	Zhao C. et al. (2015)
		heat shock protein 70	Zhao C. et al. (2015)
		heat shock protein 83	Zhao C. et al. (2015)
		heat shock protein 90	Zhao C. et al. (2015)
3	Mechanical stimulus	Vacuolar H(+) - ATPase subunit A	Zhao C. et al. (2015)
		Vacuolar H + −ATPase B subunit	Zhao C. et al. (2015)
		Plasma membrane H + −ATPase	Zhao C. et al. (2015)
		Autoinhibited H+ ATPase	Zhao C. et al. (2015)
		Autoinhibited calcium ATPase	Zhao C. et al. (2015)
		Cytosolic ascorbate peroxidase	Zhao C. et al. (2015)
		Peroxidase	Zhao C. et al. (2015)
		Glutathione peroxidase	Zhao C. et al. (2015)
4	Hormone biosynthesis and response relative genes	Auxin conjugate hydrolase	Zhao C. et al. (2015)
		Auxin-induced-related protein	Zhao C. et al. (2015)
		Auxin-induced protein X10A	Zhao C. et al. (2015)
		Auxin-induced protein 15A	Zhao C. et al. (2015)
		Auxin-repressed protein	Zhao C. et al. (2015)
		Auxin-responsive GH3 product	Zhu et al. (2014)
		Auxin binding protein 1	Zhu et al. (2014)
		Auxin response factor 4	Zhu et al. (2014)
		Auxin response factor GTPase activator	Zhu et al. (2014)
		Auxin efflux carrier	Zhu et al. (2014), Zhao C. et al. (2015)
		Auxin efflux carrier protein 2	Zhu et al. (2014)
		Auxin efflux carrier component	Zhu et al. (2014)
		Gibberellin 20-oxidase	Zhu et al. (2014)
		Gibberellin 2-oxidase	Zhu et al. (2014)
		Gibberellin 3-oxidase	Zhu et al. (2014)
		Gibberellin regulated protein	Zhu et al. (2014)
		Gibberellin receptor GID1	Zhu et al. (2014)
		GA-like protein	Zhu et al. (2014)
		Ethylene-responsive transcription factor 1A	Zhu et al. (2014)
		Ethylene-overproduction protein	Zhu et al. (2014)
		Ethylene-responsive transciptional coactivator-like protein	Zhu et al. (2014)
		Ethylene-forming-enzyme-like dioxygenase	Zhu et al. (2014)
		ABA 8′-hydroxylase	Zhu et al. (2014)
		ABA-glucosyltransferase	Zhu et al. (2014)
		Brassinosteroid biosynthetic protein LKB	Zhu et al. (2014), Zhao C. et al. (2015)
		Brassinosteroid insensitive 1-associated receptor kinase 1	Xia et al. (2013)
		Brassinosteroid receptor	Zhao C. et al. (2015)
		Brassinazole resistant 1 protein	Zhao C. et al. (2015)
5	Embryonic development relative genes	Embryo-abundant protein EMB	Zhu et al. (2014)
		Seed maturation protein PM39	Zhu et al. (2014)
		Late embryongenesis abundant protein	Zhu et al. (2014)
		51 kDa seed maturation protein	Zhu et al. (2014)
		Ripening related protein	Zhu et al. (2014)
		Late embryogenesis abundant protein 2	Zhu et al. (2014)
		alpha-trehalose-phosphate synthase	Zhu et al. (2014)
6	Embryo abortion and Cell apoptosis relative genes	Dead box ATP-dependent RNA helicase	Zhu et al. (2014)
		Lethal leaf spot 1-like protein	Zhu et al. (2014)
		Senescence-associated nodulin 1A	Zhu et al. (2014)
		Senescence-related protein	Zhu et al. (2014)
		Lethal leaf spot 1-like protein	Zhu et al. (2014)
		Vascular associated death 1	Zhu et al. (2014)
		Leucine-rich repeat	Zhu et al. (2014)
		NB-LRR type disease resistance protein Rps1-k-1	Zhu et al. (2014)
		NB-LRR type disease resistance protein Rps1-k-1	Zhu et al. (2014)
		R 8 protein	Zhu et al. (2014)
		R 10 protein	Zhu et al. (2014)
		candidate resistance protein KR1	Zhu et al. (2014)
		Putative senescence-associated protein	Zhu et al. (2014)
		Late embryogenesis-abundant protein group 9 protein	Chen et al. (2013)
7	Energy and carbohydrate metabolism	Fructose bisphosphate aldolase	Zhu et al. (2013)
		Triosephosphate isomerase	Zhu et al. (2013)
		putative stearoyl-acyl carrier protein desaturase	Zhu et al. (2013)
		Class III endochitinase	Zhu et al. (2013)
8	Ca^2+^ regulation	CAM5 (calmodulin 5); calcium ion binding	Zhao C. et al. (2015), Yang et al. (2017)
		Calcium-binding protein	Zhao C. et al. (2015)
		Endoplasmic reticulum-type calcium-transporting ATPase 4	Zhao C. et al. (2015)
		OST1 (open stomata 1); calcium-dependent protein serine/threonine kinase protein serine/threonine kinase	Zhao C. et al. (2015)
		Calcium-binding EF hand family protein	Zhao C. et al. (2015)
9	Protein catabolic process	Peptidyl-prolyl cis-trans isomerase	Zhu et al. (2013)
		threonine endopeptidase	Zhu et al. (2013)
		Polyubiquitin 1	Zhu et al. (2013)
		ubiquitin carrier-like protein	Zhu et al. (2013)
		Proteasome subunit alpha type-7	Zhu et al. (2013)

### The Role of Hormone Signaling Genes

Upon soil penetration, the expression of multiple genes related to auxin homeostasis and transport is affected, including those encoding tryptophan synthase, ARF, auxin-repressed protein, auxin-induced protein, auxin efflux/influx carrier proteins and auxin conjugate hydrolase (Xia et al., 2013). In later stages, growth and expansion of peg dramatically reduces because GA biosynthesis is negatively regulated in the dark due to upregulation of *DELLA* and *GA* *_2_* *oxidase*. Expression of ethylene biosynthetic genes is known to be regulated through BR (De Grauwe et al., 2005; Wei et al., 2015; Zhu et al., 2016; Zhang et al., 2016). The subterranean peg displays significantly reduced expression of the genes encoding for brassinosteroid receptor and BRASSINOSTEROID INSENSITIVE 1-associated receptor kinase 1, with increased level of gene transcripts involved in ethylene biosynthesis (Xia et al., 2013), which could be linked to the triple response phenotype of subterranean peg. Interestingly, together with BR and ethylene, auxin is known to be involved in the tripartite control of hypocotyl growth (De Grauwe et al., 2005). In the dark, *Arabidopsis* dark grown *bzr1* mutant seedlings exhibit reduced hypocotyl length and reduced sensitivity to IAA (Zhou et al., 2013). Interestingly, BZR1 (involved in BR signaling) is a dual function TF and can either activate or repress BR signaling (Wang et al., 2002). Additionally, auxin-resistant mutant *iaa19* shows insensitivity to brassinosteroids, and *ARF7* and *IAA19* gene promoters contain binding regions for the BZR1 TF (involved in BR signaling); thereby serving as direct targets for BZR1 (Zhou et al., 2013). Furthermore, BR perceived through BZR1 activates physical interaction between BZR1 family TFs and the DELLA protein, which concomitantly affects hormone and light signaling pathways (Wang et al., 2013). Darkness induces loss of flavonoids *via* reduced expression of gene *chalcone synthase* (*CHS*), a key enzyme in the flavonoid biosynthesis pathway (Xia et al., 2013), which facilitates lignin development in the developing pod *via* diverting substrate to lignin biosynthesis.

### New Players – Role of Heat Shock Proteins

Heat shock proteins (HSPs) are broadly known to be involved in stress responses; however, their roles in gravitropic responses are less explored. In *Arabidopsis*, evidence for HSPs mediated gravitropism comes from a *rha1* mutant that shows resistance to auxin and exhibits altered root bending in response to a gravitopic stimulus (Fortunati et al., 2008). A recent study has uncovered a new function of HSPs as transcription factors, therefore, they play a significant role in various processes including phosphorylation of proteins as well as interaction with calmodulin — a calcium signaling protein (Park and Seo, 2015). In peanut, transcriptome and proteomic analysis of aerial and subterranean peg revealed the expression of more than 100 HSPs-related unigenes associated with gravitropic response, mechanical stimulus and light and dark regulation. Among these, 13 HSPs were proposed to be key controllers of gravitropic response (Xia et al., 2013; Zhao C. et al., 2015). These results provide new insights into peanut biology as HSPs are also known to be associated with embryo abortion (Fu et al., 2002; Hsu et al., 2010). The role of HSPs in embryo development was demonstrated in maize and *Arabidopsis* (Fu et al., 2002; Hsu et al., 2010). In peanut, HSP-70 is highly expressed in the aerial peg, while its expression is attenuated in the subterranean peg during swelling and pod formation (Xia et al., 2013). Thus, there is scope to study and confirm if the association of HSPs with embryo abortion in peg is similar to previous findings reported in the maize and *Arabidopsis*. Recently, protein modifying- and degrading enzyme threonine endopeptidase was reported in the developing peg and pod, suggesting its critical role during embryo development and potentially premature embryo abortion (Zhu et al., 2013). In barley, threonine endopeptidase causes degeneration of aleurone cells (Swanson et al., 1998); whereas, it is associated with floral organ senescence in daylily (*Hemerocallis* spp) (Guerrero et al., 1998), thus plays a central role in the developmentally associated programmed cell death (Beers et al., 2000). Hence, further detailed studies in this regard would be useful to achieve high yield *via* improved pegging and low embryo degeneration.

## Other Regulators of Subterranean Peg and Pod Development

The growth and elongation of subterranean pod is consistent with the upregulation of genes, which facilitate cell elongation. For instance, expression of gene *GA* *_20_* *oxidase* greatly reduces, which was corroborated with the gravitropism responsive genes. At later stages, pod swelling involved downregulation of cell wall biosynthetic genes including *cellulose synthase* (Xia et al., 2013; Chen et al., 2015). Notably, mutation in *cellulose synthase* results in cell swelling (Hernández-Blanco et al., 2007). The Ca^2+^ signal transduction pathways is also important during pegging because its deficiency in soil can lead to early embryo abortions. In peanut, it has been suggested that Ca^2+^ deﬁciency affects peg swelling and pod formation *via* embryo abortion (Yang et al., 2017). Indeed, the peanut gene, *AhCYP707A4A* has been characterized to control embryo abortion by Ca^2+^ deﬁciency (Chen et al., 2015). Further, pod development involves Ca^2+^ signal transduction genes encoding CaM-binding protein, CDPK, and MAPKKK, which are probably under strong control of MADS-box family TFs, and hormones — GA and IAA (Yang et al., 2017). Additionally, five calcium-related proteins have been identified i.e., calmodulin 5, calcium-binding EF hand family protein, calcium-dependent protein serine/threonine kinase, calcium-binding protein, and endoplasmic reticulum-type calcium-transporting ATPase 4, which could be responsible for the growth and development of subterranean peg (Zhao C. et al. 2015). The recent work of Yang et al. (2017) represented orchestration of gene expression of 53 Ca^2+^ related genes, along with 84 genes, which were involved in hormone signaling and biosynthesis during peg development. Further, role of Ca^2+^ signaling is also demonstrated in gravitropic response. Notably, calcium and calmodulin (CaM) play crucial role in gravity perception and signal transduction. It has been shown that SAURs (an auxin-responsive gene family) encode for Ca^2+^/CaM-binding proteins, which are also involved in gravity response (Yang and Poovaiah, 2000). In Arabidopsis, under hyper gravity and simulated micro-gravity conditions, the expression of genes involved in the Ca^2+^/CaM signaling pathway was significantly altered inducing gravitropic bending. These findings support the hypothesis that peg gravitropic response is under strong regulation of auxin signaling and also linked to Ca2+ ion signaling. Additionally, subterranean peg response to darkness and mechanical stimulus involves expression of wide range of TFs factors such as AP2-EREBP, B3, basic leucine zipper, BEL1-like, bZIP, CBF/DREB, C3HL domain class CCAAT-binding, ethylene-responsive TFs, MYB, MADS-box, NAC, GRAS, and WRKY, which in turn influence expression of hormone signaling genes (Chen et al., 2013; Xia et al., 2013).

## microRNA in Control Of Gene Expression Involved In Molecular Pathways

miRNAs play pivotal role during plant development and strategic adaptation to adverse situations. Generally, miRNAs attenuate translation of target gene transcript or can cause the cleavage of target gene transcripts. In fact, the impact of miRNAs on growth and development is much bigger than it is acknowledged, and extends far beyond post-transcriptional gene regulation or translational repression. Even though the study of miRNAs in peg development is still in early stages, their role in peg gravitropism cannot be ignored.

### microRNAs (miRNAs) Control Genes Involved in Various Pathways

Recent research in peanut demonstrated that peg and seed development are also regulated by miRNAs (Zhao et al., 2010; Chi et al., 2011; Shen et al., 2016; Gao et al., 2017; Ma et al., 2018). Peg development involves expression of several miRNAs, which are known to target different classes of genes such as transcription factors —* GRAS, SPL, TCP, MYB, NAC*, etc.; hormone signaling and sugar metabolism genes — *DELLA, ARFs, GRF*, brassinosteroid receptor kinase, F-box protein, myo-inositol-1-phosphate synthase, cellulose synthase, etc. (Gao et al., 2017). In peg, miRNAs ahy-miR171 and ahy-miR319 target *DELLA* and *GRAS* TFs, respectively. DELLA protein is a key negative regulator of the GA-signaling pathway, which is known to be involved in gravitropism. DELLA protein interacts with INDETERMINATE DOMAIN (IDD) and SCARECROW-LIKE 3 (SCL3) (Yoshida et al., 2014), which are known to play important roles in the shoot gravitropism (Tanimoto et al., 2008; Wu et al., 2013). In *Arabidopsis*, a *SGR5* gene encodes for *shoot gravitropism5* gene — a member of IDD zinc finger protein family, and loss-of-function *sgr5* mutants cause altered gravity sensing in shoot due to altered amyloplast distribution in epidermal layer of shoot (Tanimoto et al., 2008). Further, SCL3 belongs to the GRAS TF family known to regulate DELLA and its own expression through DELLA-SCL3 mediated feedback loop (Yoshida et al., 2014).

Peg elongation and pod development is also under control of miRNAs through hormone homeostasis, which involves auxin and GA (Shen et al., 2016; Gao et al., 2017). For instance, ahy-miR160, ahy-miR167 and miR390 target ARFs and ahy-miR393 target auxin signaling F-box protein (Shen et al., 2016; Gao et al., 2017). Additionally, ahy-miR390 can supress expression of *ARF* genes indirectly *via* formation of ta-siRNAs (Fahlgren et al., 2006). Some evidences come from the miRNA- ahy-miR482 and ahy-miR9666, together these are involved in ubiquitin-mediated proteolysis pathway *via* targeting E3 ubiquitin ligase gene which control the accumulation of AUX and DELLA protein.

Light is a key regulator of developmental processes including embryogenesis (Azari et al., 2010). Interestingly, the process of embryogenesis involves miR390 mediated *ARF* transcript cleavage, and miR167 and miR1088 mediated cleavage of *PPRP* transcript (Lu et al., 2011; Sauer et al., 2013). In peanut, light inhibits development of embryo and it is arrested until peg perceives a mechanical stimulus and permanent darkness in the subterranean condition (Zamski and Ziv, 1976; Ziv, 1981; Chen et al., 2016b). Expression of these miRNAs during peg development and embryogenesis supports the existence of light signaling and its interaction with multiple hormone pathways (Gao et al., 2017).

### miRNAs Target Key TFs Involved in Fruit Development and Maturation

During peanut pegging, several miRNAs target TFs that are known to be key regulators of fruit development — including both fleshy and dry fruits (Seymour and Granell, 2014). In peanut peg, ahy-miR172 targets *AP2-ERF*, ahy-miR156/157 targets *SQUAMOSA* promoter binding protein-like, ahy-miR164 target *NAC*, ahy-miR391 target *HD-ZIP* and ahy-miR396 target *MADS-box*. Reportedly, in fleshy fruits, AP2/ERF, MADS-box and NAC TFs are key regulators of metabolism, fruit development and senescence, and hormone including ethylene, by regulating genes such as *ACO, ACS, PG, EXP*, etc., (Kumar et al., 2017; Kumar et al., 2018). Notably, *AP2-ERF.B3-SRDX* stimulates expression of *RIN* (encodes MADS-box TF), *NAC-NOR* (encode NAC domain protein), *CNR* (encodes a SQUAMOSA promoter binding protein-like, target of miR156/157) and *HD-ZIP*. Further, the role of the AP2/ERF TF has been widely surveyed in several plants such as rice, *Arabidopsis*, tobacco, tomato, etc., and is known to be involved in auxin, ABA, cytokinin, ethylene, gibberellic, and jasmonate mediated development (Gu et al., 2017). Similarly, ahy-miR156 cleaves *SPL*, and subsequently decreases the miR164-mediated cleavage of *NAC* during early pod development. Importantly, miR159 targets *MYB*, which regulates the flavonol biosynthetic pathway’ including anthocyanin accumulation, fruit development and seed size in plants (Zhang et al., 2013a; Huang et al., 2016), and thus suggesting miR159 may play a role in regulating both — anthocyanin content of peg and pod development. Although, in peanut, detailed investigations into the miRNAs is currently greatly lacking, nevertheless, we expect that the approaching decade will witness focused research towards characterization of specific miRNAs and their association with development of peg and pod.

## Conclusion

New technological advancements have facilitated comprehensive studies in peanut leading to better understanding of genome size and its structure, genome-wide landscape for gene expression, and proteome dynamics during growth and development of plant. The advances also provide deeper insights on multiple aspects of peg biology including peg gravi-sensing mechanisms involving hormones and secondary messengers, hormonal- and non-hormonal regulation, photo- and skoto-regulation, gene discovery and annotation, epigenetic modifications, gene expression, proteome mapping, etc. and integration of these omics components. Peg is a key component to peanut reproduction and we believe that auxin, ABA and phytochromes play an important role in peg geotropism, and therefore, a deeper understanding in this area will enhance peanut yield. Additionally, the peanut peg can be used as model to study geotropism in plants as most of the previous studies have utilized *Arabidopsis* as a model plant. Even though the transcriptomic and proteomic studies were available in peanut; the majority of the studies were conducted in the absence of reference genome of peanut cultivated tetraploid species; thereby affecting the translation of these information in crop improvement. The use of bio-informatics tools and updated annotation of previously identified key genes will not only decipher the role in peg geotropism, but also help plant molecular biologist to add new layers to the existing knowledge of the geotropism in plants. Further, the utilization of advanced and widely used genome editing technology for dissecting genes associated with peg geotropism will help plant molecular biologist to add new layers to the existing knowledge of the geotropism in plants.

## Author Contributions

RV and RK discussed, conceptualized and designed the review’s outline. RK wrote the manuscript, and MP, SR, HN, SK, and RV edited the MS.

## Conflict of Interest

The authors declare that the research was conducted in the absence of any commercial or financial relationships that could be construed as a potential conflict of interest.
